# Individual hygiene behaviors during the COVID-19 pandemic

**DOI:** 10.1590/1980-220X-REEUSP-2022-0283en

**Published:** 2023-01-06

**Authors:** Ayla İrem Aydın, Derya Akça Doğan, Ayşe Serpici, Meryem Atak

**Affiliations:** 1 Bursa Uludag University, Faculty of Health Sciences, Department of Pediatric Nursing, Bursa, Turkey.; 2 Bursa Uludag University, Faculty of Health Sciences, Department of Medical Nursing, Bursa, Turkey.; 3 Bursa Uludag University, Faculty of Health Sciences, Department of Surgical Nursing, Bursa, Turkey.

**Keywords:** Behavio, Covid-19, Hygien, Nursin, Security Measures, Comportamento, Covid-19, Higiene, Enfermagem, Segurança, Conducta, Covid-19, Higiene, Enfermería, Medidas de Seguridad

## Abstract

**Objective:**

To investigate the hygiene behaviors of individuals during the Coronavirus Disease-2019 (COVID-19) pandemic.

**Method:**

During the pandemic (April 2021–September 2021), 439 adults were surveyed online via Google Forms, which assessed the individuals’ introductory information and hygiene behaviors as determined by the COVID-19 Hygiene Scale.

**Results:**

Out of the participants, 71.3% were female and 68.3% were 18–33 years old. The mean COVID-19 Hygiene Scale score of the participants was 94.62 ± 26.56. Individuals belonging to the 18–33 years age group had significantly higher hand hygiene scores than the other age groups (p < 0.05). Women showed a higher mean total and subdomain scores in the COVID-19 Hygiene Scale than men. A significant difference between the social distance and mask use and hand hygiene subdomains was observed (p < 0.05).

**Conclusion:**

The hand hygiene scores of the individuals varied by age and gender, whereas the social distance and mask use scores varied by only gender. Based on the above mentioned results, the nurses should focus on the individuals’ development of effective hygiene behaviors, and schedule and implement trainings according to the sociodemographic differences among the individuals.

## RESUMO

**Objetivo:**

Investigar os comportamentos de higiene dos indivíduos durante a pandemia da Doença Coronavírus-2019 (COVID-19).

**Método:**

Durante a pandemia (entre abril de 2021 a setembro de 2021), 439 adultos foram entrevistados on-line por médio do Google Forms, que avaliou as informações introdutórias e os comportamentos de higiene dos indivíduos, de acordo a Escala de Higiene COVID-19.

**Resultados:**

71.3% dos participantes eram do sexo feminino e 68.3% tinham entre 18 e 33 anos. A pontuação média dos participantes de acordo a Escala de Higiene COVID-19 foi de 94.62 ± 26.56. Indivíduos pertencentes à faixa etária de 18 a 33 anos apresentaram escores de higiene das mãos significativamente mais altos do que as outras faixas etárias (p < 0.05). As mulheres apresentaram pontuações médias totais e de subdomínios mais altas na Escala de Higiene COVID-19 do que os homens. Observou-se diferença significativa entre os subdomínios distância social e uso de máscara e higiene das mãos (p < 0.05).

**Conclusão:**

Os escores de higiene das mãos dos indivíduos variaram por idade e sexo, enquanto os escores de distância social e uso de máscara variaram apenas por gênero. Com base nos resultados referidos acima, os enfermeiros devem se concentrar em desenvolvimento de comportamentos de higiene eficazes nos indivíduos, agendar e implementar treinamentos de acordo com as diferenças sociodemográficas entre os indivíduos.

## RESUMEN

**Objetivo:**

Investigar las conductas de higiene de las personas durante la pandemia de la Enfermedad por Coronavirus 2019 (COVID-19).

**Método:**

Durante la pandemia (Abril de 2021–Septiembre de 2021), se encuestó en línea a 439 adultos a través de Formularios de Google, que evaluaron la información introductoria y las conductas de higiene de las personas como lo determine el COVID-19 Hygiene Scale.

**Resultados:**

De los participantes, el 71.3% eran mujeres y el 68.3% tenían entre 18 y 33 años. La puntuación media del COVID-19 Hygiene Scale de los participantes fue de 94.62 ± 26.56. Los individuos pertenecientes al grupo de edad de 18–33 años tuvieron puntuaciones de higiene de manos considerablemente más altas que los otros grupos de edad (p < 0.05). Las mujeres mostraron una puntuación media total y de subdominio más alta que los hombres en el COVID-19 Hygiene Scale. Se observó una diferencia importante entre la distancia social y los subdominios de uso de mascarilla e higiene de manos (p < 0.05).

**Conclusión:**

Las puntuaciones de higiene de manos de los individuos variaron por edad y sexo, mientras que las puntuaciones de distancia social y uso de mascarillas variaron solo por género. Basado en los resultados mencionados, las enfermeras deben enfocarse en el desarrollo de conductas efectivos de higiene de los individuos, y programar e implementar capacitaciones de acuerdo con las diferencias sociodemográficas entre los individuos.

## INTRODUCTION

The World Health Organization (WHO) designated the Coronavirus Disease-2019 (COVID-19) as a pandemic on March 11, 2020^(1)^. Coronavirus is transmitted via respiratory droplets and contact and has an adverse effect on human health. The strongest and most effective measure against this pandemic, which not only affected our health but also the economy, politics, and social order of our society, is the prevention of its proliferation^(2)^. Studies have been conducted on the possible pharmacological interventions for the prevention and treatment of the virus^(3)^, and guidelines have been published on nonpharmacological protective procedures as well. In the light of these guidelines, individuals are expected to improve their hygiene behaviors, wear masks, and comply with social distancing to protect themselves and others from the virus^(4,5,6)^.

The hygiene behaviors, consumption habits, and domestic activities of individuals have changed during the pandemic with the common objective of ensuring improved hygiene. to protect themselves from the disease^(7)^.

Relevant studies in the literature have explored the hand hygiene behaviors^(8,9,10)^, use of masks^(11,12)^, and compliance with social distancing^(13)^ in individuals as a protective measure against COVID-19. Furthermore, studies have collectively explored hand hygiene, mask use, and social distancing behavior in individuals^(14,15)^. However, to the best of our knowledge, few studies in the literature have comprehensively investigated the personal and general hygiene behaviors of individuals. The present study aimed to investigate the hygiene behaviors of individuals during the COVID-19 pandemic.

## METHODS

### Design of Study

This study was a descriptive, cross-sectional study of conducted through a self-administered questionnaire.

### Population

The sample size of the study was determined by the Open Epi program. The calculations by Open Epi program concluded that 375 people were to be included in the study by setting the expected value at a confidence interval of 95% in accordance with a previous, similar study (57.7%). This number was aimed to be reached as a minimum.

### Local

This study was conducted adult individuals residing in Turkey.

### Selection Criteria

The inclusion criteria for the study included being 18 years or above and agreeing to participate in the study.

### Data Collection

An online questionnaire form (Google Forms) was employed to gather information from the participants, which could be accessed upon clicking a link. The questionnaire was disseminated by the researchers using social media tools, such as Facebook, WhatsApp, and Instagram. The questionnaire dissemination occurred through personal social networks by all researchers. The survey was released every week. Incomplete survey response not recorded. To participate in the study, it was necessary to answer all the questions. The questionnaire form consisted of the contact information of the researchers, the purpose of the study, the manner of usage and privacy of the study data, and a statement for obtaining the voluntary consent of the participants.

The instrument with the introductory questions and the COVID-19 Hygiene Scale comprised a total of 37 items. The response time was 15–20 minutes. The study data were collected between April 2021 and September 2021.

Ten items in the questionnaire aimed to collect introductory information about the participants (age, gender, marital status, COVID-19 diagnosis status etc.) as developed by the researchers considering the relevant literature, and the 27-item COVID 19 Hygiene Scale determined the hygiene behaviors of the individuals. The COVID-19 Hygiene Scale was developed by Çiçek et al.^(7)^ with an aim to determine the changing behaviors of individuals with regard to hygiene during the pandemic. The Cronbach’s Alpha internal consistency coefficient of the scale was .908, and thus, the scale was deemed to be highly reliable. In the present study, the Cronbach’s Alpha internal consistency coefficient was .969.

The abovementioned scale consisted of six subdomains that included changing hygiene behaviors in the pandemic, home hygiene, social distancing and mask use, shopping hygiene, hand hygiene, and hygiene when coming home from outside. The scale used a 5-point rating system (from always, 5 points to never, 1 point). The lowest possible score on the scale is 27, whereas the highest possible score is 135. The lowest and highest possible scores from the subdomains of the scale are as follows: changing hygiene behaviors, home hygiene, social distancing and mask use, shopping hygiene, hand hygiene and hygiene when coming home from outside. A high score indicated that the a high level of personal and general hygiene behavior of the individual and more attachment to the importance of hygienic measures with an aim to protect themselves from the pandemic^(7)^.

### Data Analysis and Treatment

Numerical and percentage distributions were used in the data pertaining to the sociodemographic features and COVID-19 Hygiene Scale scores of the participants. The data was tested for normal distribution using the Shapiro–Wilk test. As per the results of the normality test, Mann–Whitney U and Kruskal–Wallis tests were used for intergroup comparisons, and Bonferroni correction was made subsequent to the pairwise comparisons. The Statistical Package for Social Sciences Version 23.0 software package was used in the analysis of the data.

### Ethical Aspects

The ethical approval for the study was obtained from the Health Sciences Research and Publication Ethics Committee (Decision no: 2021-03). The written permission from the responsible author for the use of the COVID-19 Hygiene Scale was obtained via e-mail.

## RESULTS

The total of 439 participants were included in the research. The mean participant age was 31.47 ± 10.82 (18–64) years and 68.3% of the participants were included in the 18–33 age group; 71.3% were female, 57.4% were single, and 46.0% were college graduates. Individuals with a self-reported income level of moderate constituted 73.6% of the group (Table 1).

**Table 1.** COVID-19 Hygiene Scale subdomain scores based on introductory information of participants – Bursa, Marmara Region, Turkey, 2021.

COVID-19 Hygiene Scale								
	n(%)	SD1	SD2	SD3	SD4	SD5	SD6	Total
Age								
18–33	300(68.3)	20.13 ± 5.67	13.51 ± 4.05	15.96 ± 4.18	15.95 ± 5.83	20.15 ± 5.13	10.35 ± 3.23	96.06 ± 24.85
34–49	95(21.6)	19.64 ± 6.26	12.90 ± 4.30	15.43 ± 4.47	16.31 ± 5.77	18.89 ± 5.59	9.60 ± 3.47	92.78 ± 27.36
>50	44(10)	18.79 ± 7.59	13.00 ± 5.25	14.47 ± 5.27	15.72 ± 7.38	17.63 ± 6.53	9.18 ± 4.20	88.84 ± 34.63
	*p* value	0.561	0.543	0.340	0.866	0.030	0.062	0.556
	KW^1^	1.156	1.221	2.156	0.289	7.009	5.551	1.174
Sex								
Female	313(71.3)	20.12 ± 5.80	13.52 ± 4.00	16.12 ± 4.18	16.19 ± 5.86	20.01 ± 5.34	10.19 ± 3.35	96.18 ± 25.61
Male	126(28.7)	19.31 ± 6.50	12.84 ± 4.75	14.62 ± 4.67	15.54 ± 6.26	18.65 ± 5.58	9.76 ± 3.53	90.76 ± 28.5
	*p* value	0.252	0.178	0.030	0.306	0.011	0.257	0.083
	Z^2^	–1.146	–1.346	–2.940	–1.023	–2.542	–1.132	–1.733
Marital status								
Single	252(57.4)	20.26 ± 5.81	13.31 ± 4.12	15.78 ± 4.29	15.69 ± 5.85	20.02 ± 5.25	10.21 ± 3.38	95.30 ± 25.31
Married	187(42.6)	19.39 ± 6.26	13.34 ± 4.39	15.58 ± 4.50	16.42 ± 6.14	19.08 ± 5.66	9.88 ± 3.44	93.72 ± 28.20
	*p* value	0.195	0.812	0.950	0.199	0.107	0.311	0.810
	Z^2^	–1.297	–0.238	–1.285	–1.285	–1.613	–1.013	–0.240
Graduated education level								
Literate/Primary	23(5.3)	19.82 ± 7.46	13.86 ± 5.23	13.78 ± 5.55	15.34 ± 6.80	16.56 ± 6.19	9.21 ± 4.38	88.60 ± 34.23
Middle/High	96(21.9)	20.20 ± 6.56	13.81 ± 4.61	15.20 ± 4.91	16.36 ± 6.73	18.5 ± 6.04	10.20 ± 3.53	94.31 ± 30.13
Associate degree	59(13.4)	20.54 ± 6.44	13.72 ± 4.66	16.05 ± 4.43	16.88 ± 6.13	19.49 ± 5.68	10.23 ± 3.47	96.93 ± 28.76
College degree	202(46)	19.85 ± 5.77	12.99 ± 3.90	15.79 ± 4.25	15.81 ± 5.66	20.1 ± 5.25	10.06 ± 3.32	94.69 ± 24.96
Higher degree	59(13.4)	18.91 ± 4.81	13.10 ± 3.86	16.55 ± 2.87	15.47 ± 5.29	20.84 ± 3.61	10.05 ± 3.10	94.94 ± 19.74
	*p* value	0.746	0.183	0.333	0.160	0.478	0.819	0.716
	KW^1^	1.231	4.857	3.407	10.305	1.4750	0.925	1.356
Economic status								
Income more than expense	77(17.5)	20.12 ± 4.76	13.63 ± 3.97	16.76 ± 3.52	16.25 ± 5.56	20.75 ± 3.88	10.24 ± 3.09	97.79 ± 22.23
Income equals the expense	323(73.6)	20.01 ± 6.12	13.36 ± 4.17	15.61 ± 4.31	15.97 ± 6.05	19.54 ± 5.47	10.06 ± 3.44	94.58 ± 26.70
Income less than expense	39(8.9)	18.41 ± 7.23	12.41 ± 5.18	14.25 ± 5.84	15.79 ± 6.27	18.07 ± 7.26	9.76 ± 3.79	88.71 ± 33.65
	*p* value	0.478	0.489	0.116	0.919	0.586	0.893	0.670
	KW^1^	1.475	1.431	4.304	0.170	1.068	0.227	0.801

^1^Kruskal–Wallis test; ^2^Mann–Whitney U test; SD: Sub-dimension; SD1: Changing hygiene behaviors in the pandemic; SD2: Home hygiene; SD3: Social distancing and mask use; SD4: Shopping hygiene; SD5: Hand hygiene; SD6: Hygiene when coming home from outside.

Table 1 shows the total and subdomain mean scores of the introductory information of the study participants. A significant difference was observed in the hand hygiene subdomain in the participants belonging to the 18–33 and >50 age groups (p < 0.05; Bonferroni correction was used). The mean hand hygiene subdomain score of the 18–33 age group was higher than that of >50 age group. Although the mean scores of all the subdomains of the scale and the total mean score of the 18–33 age group were higher than the other two age groups (34–59 and >50 years), no significant difference was observed in any of the subdomains among the different age groups other than in the hand hygiene subdomain (p > 0.05, Table 1).

Compared to the male participants, the female participants had a higher mean total score and mean scores of all the subdomains of the COVID-19 Hygiene Scale. Further, a significant difference was found between the social distancing and mask use and hand hygiene subdomains in the female participants (p < 0.05). No significant difference among the education, marital status, and income level of the participants and their COVID-19 Hygiene Scale and its subdomains’ scores was observed (p > 0.05, Table 1).

The features of the participants regarding COVID-19 have been provided in Table 2. While 57.9% of the respondents were employed before the COVID-19 pandemic, the rate of individuals who worked at a work site was 31.9% during the pandemic. As per the questionnaire responses, 23.0% of the participants and 42.1% of the participants’ family members were COVID-19 positive (Table 2).

**Table 2.** COVID-19-Related Features of Participants – Bursa, Marmara Region, Turkey, 2021.

	n (439)	%
Worked status before COVID-19*		
Yes No	254 185	57.9 42.1
Worked status in the COVID-19*		
Not worked	194	44.2
Worked at a home	37	8.4
Some days worked at a home	68	15.5
Worked at a work site	140	31.9
**Diagnosed with COVID-19**		
Yes No	101 338	23.0 77.0
**Diagnosed with COVID-19 in the family**		
Yes No	185 254	42.1 57.9

*Before March 11, 2020.

No significant difference was observed between the total scale and subdomain scores in the following subdomains: employment status before COVID-19, place of work during COVID-19, being diagnosed with COVID-19, and having family members who were diagnosed with COVID-19 (p > 0.05). Nevertheless, the COVID-19 Hygiene Scale mean score was higher in the participants who were diagnosed with COVID-19 or had family members who were diagnosed with COVID-19 (Table 3). The total mean score of the COVID-19 Hygiene Scale of the participants was 94.62 ± 26.56.

**Table 3.** COVID-19 Hygiene Scale subdomain scores based on COVID-19-related features – Bursa, Marmara Region, Turkey, 2021.

	COVID-19 Hygiene Scale						Total
	SD1	SD2	SD3	SD4	SD5	SD6	
Worked status before COVID-19							
Yes	20.17 ± 6.10	13.51 ± 4.33	15.90 ± 4.29	16.45 ± 5.92	20.01 ± 5.27	10.23 ± 3.35	96.30 ± 26.33
No	19.51 ± 5.89	13.08 ± 4.10	15.41 ± 4.48	15.38 ± 6.02	19.08 ± 5.64	9.84 ± 3.48	92.32 ± 26.77
*p* value	0.281	0.247	0.205	0.067	0.094	0.27	0.133
Z^2^	–1.078	–1.158	–1.268	–1.883	–1.674	–1.102	–1.501
Working status in the COVID-19*							
Not worked	19.58 ± 6.30	13.35 ± 4.42	15.34 ± 4.68	15.76 ± 6.26	18.95 ± 5.93	9.94 ± 3.68	92.94 ± 28.73
Worked at a home	21.24 ± 5.40	14.02 ± 3.78	16.18 ± 3.94	17.51 ± 5.22	20.02 ± 5.12	10.51 ± 2.67	99.51 ± 22.80
Some days worked at a home	2.27 ± 5.42	13.13 ± 3.78	16.29 ± 3.64	16.39 ± 5.20	20.25 ± 4.30	10.23 ± 3.01	96.58 ± 21.74
Worked at a work site	19.77 ± 6.04	13.21 ± 4.31	15.76 ± 4.37	15.75 ± 6.20	20.15 ± 5.25	10.05 ± 3.39	94.72 ± 26.50
*p* value	0.368	0.649	0.741	0.284	0.542	0.825	0.568
KW^1^	2.00	0.863	0.598	2.516	1.227	0.385	0.568
Get COVID-19*							
Yes	19.63 ± 5.72	12.88 ± 4.31	15.56 ± 4.19	15.27 ± 6.15	19.89 ± 4.95	9.59 ± 3.47	92.84 ± 25.60
No	19.97 ± 6.11	13.46 ± 4.21	15.73 ± 4.43	16.22 ± 5.92	19.54 ± 5.58	10.21 ± 3.38	95.16 ± 26.85
*p* value	0.507	0.231	0.334	0.177	0.973	0.112	0.319
Z^2^	–0.663	–1.197	–0.966	–1.35	–0.34	–1.589	–0.997
Get COVID-19 in the family							
Yes	19.78 ± 5.85	13.17 ± 4.14	15.56 ± 4.33	15.90 ± 6.17	19.58 ± 5.38	10.00 ± 3.37	94.02 ± 26.28
No	19.97 ± 6.14	13.44 ± 4.31	15.79 ± 4.41	16.07 ± 5.84	19.65 ± 5.49	10.12 ± 3.44	95.06 ± 26.80
*p* value	0.743	0.483	0.351	0.807	0.6	0.672	0.438
Z^2^	–0.328	–0.702	–0.932	–0.245	–0.524	–0.424	–0.661

*Before March 11, 2020. ^1^Kruskal–Wallis test; ^2^Mann–Whitney U test; SD: Sub-dimension; SD1: Changing hygiene behaviors in the pandemic; SD2: Home hygiene; SD3: Social distancing and mask use; SD4: Shopping hygiene; SD5: Hand hygiene; SD6: Hygiene when coming home from outside.

## DISCUSSION

National and international guidelines published since the onset of the COVID-19 pandemic have recommended simple measures to prevent disease. These measures emphasized the need to improve individual hygiene behaviors, wear masks, and comply with social distancing^(4–6)^.

The mean COVID-19 Hygiene Scale score of the study participants was 94.62 ± 26.56, whereas in a study with 837 adults, Çiçek et al.^(7)^ reported the same score to be 112.31 ± 15.46. In the study Çiçek et al.^(7)^, the participants responded to the items as per their hygiene behaviors during the timeframe from the date the epidemic first appeared in Turkey to the date of relaxation of the measures (March 11, 2020–June 1, 2020). In this study, data were collected between April 2021 and September 2021. The lower COVID-19 Hygiene Scale mean score in the present study was considered to be associated with the partial management of the pandemic and the improvement in hygiene behaviors as a response to the pandemic.

In the present study, a significant difference was observed in the hand hygiene subdomain mean scores of the participants belonging to different age groups. The participants in the 18–33 age group had a higher hand hygiene subdomain mean score than those in the >50 age group. A study conducted with 1591 American adults investigating the perceived risk of the virus and the tendency to engage in protective behaviors did not fin hand hygiene to vary by age, unlike the results of the present study^(16)^. Also contrary to the findings of this study, Çiçek et al.^(17)^ reported that hand washing tendency increased with age. According to Mahdi et al.^(10)^, 98% of the participants of their study reported awareness about the role of hand hygiene in the prevention of COVID-19. Mieth et al.^(18)^, in their study with 1434 participants, found that 94.5% of the participants in the direct questioning group and 78.1% of the participants in the indirect questioning group practiced appropriate hand hygiene. Dwipayanti et al.^(8)^ emphasized that participants who considered hand hygiene an effective measure against COVID-19 and who perceived that they were at risk of contracting COVID-19 washed their hands more frequently. Harper et al.^(19)^ stated that the fear of contracting COVID-19 was the only positive and stable predictor of health-protective behavior. The results of these studies suggested that individuals with a higher perceived risk of contracting COVID-19 strongly considered hand hygiene in the scope of protective behaviors. The present study demonstrated that a younger age group was more proactive in ensuring hand hygiene, which might be associated with a higher prevalence of COVID-19 anxiety in this age group. This can be further explained by the wider social networks of individuals within this age group and more engagement in social contact. A meta-analysis on 277 studies with 177.635 participants investigated age-related social network changes and determined that the global social network increased until young adulthood and then steadily decreased^(20)^. Taking into consideration the various populations with different cultural, ethnic, and geographical origins, the changes in hand hygiene/protective behavior in those populations may differ from what the present study reports.

In this study, the mean scores of the hand hygiene, social distance, and mask use subdomains of the female study participants were higher than that of the male participants. Olaimat et al.^(15)^ also reported that women exhibited significantly more appropriate hygiene and social distancing behaviors than men. Zhong et al.^(21)^ suggested a relationship between lower levels of knowledge about COVID-19 in men and unsafe practices regarding the pandemic. A meta-analysis of 85 studies showed that in the general population, women were approximately 50% more likely to adopt/comply with the nonpharmacological measures against COVID-19, such as hand hygiene, mask use, and avoidance of crowds, than men^(22)^. Relevant studies in the literature reported that the rate of wearing masks as a protective measure against COVID-19 was significantly higher in women than men^(11,12)^. Similarly, Çiçek et al.^(17)^ stated that women demonstrated higher levels of both individual and general hygiene behaviors. These results further suggest that women are more inclined to exhibit effective hygiene behaviors for the protection of public health. Therefore, the abovementioned findings suggest that men should be targeted for the purposes of inducing and improving hygiene behaviors in the future.

## CONCLUSIONS

The findings of this study have potential implications for both future public health interventions to address protective hygiene behaviors and future research. The present study found that the hand hygiene scores of the participants varied by age and gender, and the social distancing and mask use scores varied by only gender. There was no significant difference in the hygiene scores as per the participants’ marital status, education level, income level, employment status, COVID-19 diagnosis. The present study contributes to a better understanding of the changing behaviors of individuals toward hygiene. It is important for individuals to sustain good hygiene behaviors for the prevention of COVID-19 proliferation. Trainings for the improvement of hygiene behavior in individuals should be planned and implemented in accordance with the recommendations of nurses.
